# Apolipoprotein CIII Is an Important Piece in the Type-1 Diabetes Jigsaw Puzzle

**DOI:** 10.3390/ijms22020932

**Published:** 2021-01-19

**Authors:** Ismael Valladolid-Acebes, Per-Olof Berggren, Lisa Juntti-Berggren

**Affiliations:** The Rolf Luft Research Center for Diabetes and Endocrinology, Karolinska Institutet, Karolinska University Hospital, Anna Steckséns gata 53, SE-171 76 Stockholm, Sweden; ismael.valldolid.acebes@ki.se (I.V.-A.); per-olof.berggren@ki.se (P.-O.B.)

**Keywords:** apolipoprotein CIII, type-1 diabetes, β-cells, apoptosis, inflammation, calcium channels

## Abstract

It is well known that type-2 diabetes mellitus (T2D) is increasing worldwide, but also the autoimmune form, type-1 diabetes (T1D), is affecting more people. The latest estimation from the International Diabetes Federation (IDF) is that 1.1 million children and adolescents below 20 years of age have T1D. At present, we have no primary, secondary or tertiary prevention or treatment available, although many efforts testing different strategies have been made. This review is based on the findings that apolipoprotein CIII (apoCIII) is increased in T1D and that in vitro studies revealed that healthy β-cells exposed to apoCIII became apoptotic, together with the observation that humans with higher levels of the apolipoprotein, due to mutations in the gene, are more susceptible to developing T1D. We have summarized what is known about apoCIII in relation to inflammation and autoimmunity in in vitro and in vivo studies of T1D. The aim is to highlight the need for exploring this field as we still are only seeing the top of the iceberg.

## 1. Introduction

Diabetes mellitus has been known for more than 3500 years, but still there are many unanswered questions. It is a heterogeneous disease, mainly divided into type-1 (T1D), consisting of 10–15% of the cases, and type-2 diabetes (T2D).

In T1D, there is a destruction of the insulin secreting pancreatic β-cells resulting in insulin deficiency. Genetic, immunological and environmental factors are involved in the pathogenesis, although it is likely that their relative contribution vary in different individuals.

T1D is an autoimmune disease and there are several biomarkers serving as risk indicators. A genetic predisposition is required and certain high-risk human leukocyte antigen (HLA) genotypes have been identified [1,2,3]. There is a genetic inheritance, but only 15% of patients with T1D have a first-degree relative with the disease.

Autoantibodies, single or multiple, can be detected years before the onset of T1D and are measurable signs of immunological activity although their pathogenic significance remains unclear. In the general population, the risk of T1D is about 0.5% and studies have found that the presence of one autoantibody increases the risk, but fewer than 10% of those with a single autoantibody develop T1D [4]. In the Environmental Determinants of Diabetes in the Young (TEDDY) study, where children with an increased genetic risk of T1D have been followed since birth, they reported a 70% risk of developing T1D within 10 years in children with two or more autoantibodies [5]. As possible trigger factors, initiating the destruction process of the β-cells, viral infections, D-vitamin, increased insulin demand, toxins, chemical compounds, intestinal microbiota are examples of what has been discussed [6]. A major problem to identify the triggering factor(s) is that, although T1D usually appears during childhood or adolescence, it can be diagnosed at any age. Another problem is that we have not had tools to in vivo measure ongoing β-cell death.

In this review, we will focus on the pro-inflammatory factor apolipoprotein CIII (apoCIII) and its possible role as a co-player in the multifaceted process that progressively destroys the pancreatic β-cells resulting in T1D.

## 2. Apolipoprotein CIII, Structure and Function

ApoCIII is a 79 amino acid glycoprotein with a molecular weight of 8.8 kDa [7,8]. ApoCIII is mainly expressed in hepatocytes and, to a lesser extent, in enterocytes [9,10,11,12]. There are three different isoforms: apoCIII_0,_ apoCIII_1_ and apoCIII_2,_ with 0, 1 or 2 sialic acid molecules bound to the protein [13,14]. The different isoforms contribute, respectively, to approximately 10, 55, and 35% of the total apoCIII levels in circulation [15]. The importance of the post-translational modification has been discussed. Mutagenesis of the glycosylation site and expression in cell lines suggest that intracellular glycosylation is not required for transport and secretion [16]. It has also been demonstrated that lack of glycosylation does not affect the binding of apoCIII to very low-density lipoproteins (VLDLs) [16]. However, nearly two decades later, it was shown that the kinetics of the isoforms apoCIII_1_ and CIII_2_ show the strongest correlation to hypertriglyceridemia and reduced VLDL, intermediate-density lipoproteins (IDLs) and apoB-100 catabolism, which are important risk factors for cardiovascular diseases (CVDs) [17]. Furthermore, the degree of sialylation affects the hepatic clearance by triglyceride-rich lipoprotein (TRL) receptors [18]. There was a similar, concentration-dependent, inhibitory effect on lipoprotein lipase (LPL) activity when total and the three isoforms of apoCIII from patients with CVD were tested separately [19]. Neither was there a difference in the increase in cytoplasmic free Ca^2+^ concentration ([Ca^2+^]_i_) upon depolarization in pancreatic β-cells exposed to the three isoforms of apoCIII [20].

ApoCIII is the most abundant C-apolipoprotein in humans and is present on TRLs, high-density lipoprotein (HDL) and low-density lipoprotein (LDL) particles [21,22,23,24,25]. ApoCIII has been defined as an important serum factor involved in lipid metabolism [23,24,26]. The main pathways by which apoCIII exerts its actions are the inhibition of lipoprotein lipase (LPL)-mediated lipolysis and the prevention of the hepatic clearance of TRL via the LDL receptor (LDLR) and LDL-related protein 1 (LRP1) [27,28,29,30,31,32]. Both mechanisms are tightly related since the clearance of circulating triglycerides (Tgs) is linked to lipolysis of TRLs by LPL [27,28,29,30,31,32].

## 3. ApoCIII Gene Regulation

The gene-encoding human apoCIII is located in a cluster between *APOAI* and *APOAIV* on chromosome 11 [33,34,35]. The cluster gene, and specifically apoCIII gene expression, is under the control of a common enhancer located 590 to 790 nucleotides upstream of the apoCIII gene [35,36,37]. There are a number of factors involved in the regulation of the gene, but glucose, insulin and cytokines are of particular interest in relation to diabetes [38,39,40,41].

### 3.1. Insulin

Under physiological conditions, apoCIII gene expression is negatively regulated by insulin, which represses the activity of the apoCIII promoter activity via inhibition of the insulin/phorbol ester responsive element (IRE) within the apoCIII gene [42,43,44]. The inhibitory action of insulin on the gene expression is mediated by the nuclear transcription factor forkhead box O1 (Foxo1), known as signal transductor of insulin for liver gluconeogenesis [38,45,46]. The sequence −498/−403 located in the apoCIII promoter, containing an IRE that mediates the repressing action of insulin on apoCIII gene expression, is also a target site for Foxo1 [38]. Thus, Foxo1 is suggested to be the responsible mediator regulating apoCIII promoter activity in response to insulin [38].

### 3.2. Glucose

In contrast to insulin, glucose increases apoCIII gene expression in rodents and humans both in vitro and in vivo [38,39]. However, for many years, the mechanisms by which glucose controls the apoCIII gene remained unknown. Glucose-mediated liver gene regulation relies on the carbohydrate response element-binding protein (ChREBP), together with the participation of other factors for the glucose response, such as the hepatocyte nuclear receptor-4α (HNF-4α) and liver X receptors (LXRs) [47,48,49,50,51,52]. The effect of glucose on apoCIII gene expression has been shown to be mediated by activation of liver pyruvate kinase (PK), ChERBP and HNF-4α [39]—the latter is required for intestinal and hepatic apoCIII expression by the interaction with its binding site in the enhancer of the apoCIII gene [53]. It is also an essential participant of the glucose response complex on the hepatic PK promoter [51].

### 3.3. Cytokines

Other factors regulating apoCIII gene expression are pro-inflammatory cytokines and signaling molecules [40,41]. The acute phase inflammatory response is mediated by cytokines such as tumor necrosis factor α (TNF-α), interleukin-1 (IL-1) and IL-6. TNF-α and IL-1 control apoCIII gene expression by repressing the promoter activity of the gene. It has been shown in vitro, that TNF-α-induced complexes are related to C/EBPδ/NF-IL6-β (CAAT enhancer-binding protein δ/nuclear factor/IL6-β) and p50 and that overexpression of C/EBPδ mimics the repressing effect of TNF-α on the promoter activation of the apoCIII gene. Additionally, it appears that the proximal and distal regulatory elements, CIIID and CIII-I, respectively, also bind to factors activated by different signaling pathways such as the nuclear factor kappa-B (NF-κB) that in a complex way, involving multiple regulatory elements, influences the apoCIII production rate [54].

## 4. ApoCIII and Inflammation

### 4.1. Vascular Effects

It has been established for many years that there is a relationship between apoCIII and CVD [55,56,57,58,59]; the nature of this is not only due to modulations in lipoprotein metabolism, but also inflammation, which is regarded as an important part of the development of atherosclerosis. ApoCIII-rich lipoproteins, as well as apoCIII itself, increase the adhesion of monocytes to vascular endothelial cells (ECs) by activation of protein kinase C-α (PKC-α), NF-κB and β1-integrins in monocytes [55,56,60]. The expression of vascular cell adhesion molecule-1 (VCAM-1) in ECs is also increased by apoCIII, thus facilitating adhesion of monocytes and thereby the development of atherogenesis [55].

Another effect of higher levels of apoCIII is an increased sialylation of the lipoprotein and it is the sialylated isoforms that can induce an increased secretion of the pro-inflammatory mediators IL-6, IL-8 and TNFα, as well as expression of intracellular adhesion molecule (ICAM-1) [61].

### 4.2. Inflammasomes

Inflammasomes are large intracellular multi-protein multimeric complexes that have the ability to integrate a number of signals from pathogen-associated molecular patterns (PAMPs), derived from invading pathogens and danger-associated molecular patterns (DAMPs) derived from endogenous stress, into a pro-inflammatory response [62]. The nod-like pyrin domain-containing 3 (NLRP3) inflammasome is the most studied and it is related to a variety of diseases and, therefore, there has been interest in finding endogenous factors that induce the sterile inflammation mediated by the inflammasome with the aim to find new therapeutic targets [63]. When serum lipoproteins were tested for their ability to induce IL-1β in human monocytes, apoCIII was identified as an activator of the NLRP3 inflammasome [63]. Interestingly, apoCIII induced an alternative inflammasome activation by heterotrimerization of Toll-like receptors 2 and 4 and the Toll-like receptor adapter protein SCIMP (SLP adaptor and CSK interacting membrane protein) [63]. These data are of importance for understanding the regulation of the NLRP3 inflammasome and thereby providing new possibilities for preventive treatment strategies.

**Figure 1:**
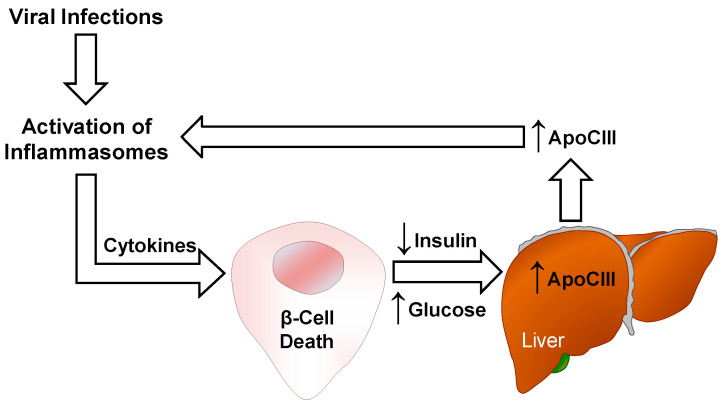
Possible mechanisms by which viral infections can induce β-cell death. Activation of inflammasomes and cytokine release upon viral infections can be participating in β-cell death leading to insulin deficiency and hyperglycemia. This results in elevated apoCIII, which mediates an alternative activation of inflammasomes.

Interestingly, a number of viruses have been associated with T1D, including enteroviruses, rotavirus, parechovirus, rubella and mumps virus. [64]. The relationship between viral infections and autoimmune diabetes is complex, involving several mechanisms, and, with the knowledge that apoCIII activates NLRP3 inflammasome, a possible contributing pathway is depicted in Figure 1.

## 5. ApoCIII and Autoimmunity

ApoCIII was discovered in 1969 [7], but little is known about whether there is a link to autoimmunity. To the best of our knowledge, this lipoprotein has only been studied in systemic lupus erythematosus (SLE) [65], primary antiphospholipid syndrome (PAPS) [66] and T1D [20,67].

SLE is a chronic inflammatory autoimmune disease. One severe manifestation, affecting the kidneys in 60% of adults and 80% of children with SLE, is lupus nephritis. Analysis of serum levels of apoCIII in controls and SLE patients with and without nephritis revealed that there was an increase in those with nephritis [63]. As atherosclerosis in SLE patients is not solely depending on traditional risk factors [68,69], the authors suggest that the increase in the pro-atherogenic apoCIII could be a contributing factor to the renal complication and might be used as a biomarker for the risk of developing nephritis and atherosclerosis [65].

PAPS is characterized by the presence of antiphospholipid antibodies and idiopathic thrombosis. Proteomic analysis of serum samples from 14 patients with PAPS and 17 sex- and age-matched controls was performed with the aim to identify proteins that could be used in the evaluation, diagnosis and prognosis of PAPS. Of 65 proteins, nine were upregulated in relation to serum from control subjects. Four of these: fibrinogen α-chain, fibrinogen γ-chain, α-1-glycoprotein-1 and apoCIII, are according to the authors functionally involved in processes associated with the induction of a procoagulant state and with autoimmune response, but to confirm the findings they conclude that more studies are necessary [66].

The serum levels of apoCIII are increased in T1D [70,71,72,73,74,75,76,77,78,79]. This can probably, to a major extent, be explained by the fact that insulin induces a dose-dependent down-regulation of the apoCIII gene at the transcriptional level [42].

Sera from a group of patients with T1D and first-degree relatives affected intracellular Ca^2+^-handling in healthy pancreatic β-cells, but this was not correlated to the presence of autoantibodies [80].

Based on the few available data, it is not possible to exclude that there can be a link between apoCIII and autoimmunity, but this needs to be further investigated.

## 6. T1D and ApoCIII

### 6.1. Serum

We have previously shown that exposing pancreatic β-cells to serum from patients with T1D increases the activity of voltage-gated Ca^2+^-channels (CaV) [81]. This leads to increased cytoplasmic free Ca^2+^ concentration ([Ca^2+^]_i_) and apoptosis. These effects can be prevented by Ca^2+^-channel blockers [81]. To identify what it was in the diabetic sera that induced the observed effects, several different fractions of sera were tested. Finally, we were able to establish apoCIII as the responsible factor and that the levels of this apolipoprotein were increased in sera from TID patients compared to healthy control subjects [20]. As a proof of concept, diabetic serum and pure apoCIII added to normal sera, with and without antisera against apoCIII, were tested and all confirmed that increased levels of apoCIII are detrimental to β-cells [20].

### 6.2. Voltage-Gated L-Type Ca^2+^ Channels

The CaV is a key player for the function of insulin-secreting cells. CaV channels are divided into low- and high-CaV channels depending on their activation thresholds. The L-type channels have a larger unitary conductance and mediate long-lasting currents (L for larger and long-lasting). In β-cells, the major type of CaV channels is the CaV1 that conducts L-type Ca^2+^ currents. The channels, located in the plasma membrane, regulate in a very strict way the influx of Ca^2+^ to the cytoplasm. Membrane depolarization changes the channels from an impermeable to a Ca^2+^ permeable state [82]. Exposing cells to T1D serum hyperactivated the subtypes CaV1.2 and CaV1.3 channels by increasing their conductivity and number [83].

Although the exact molecular mechanisms are not known, it has been demonstrated that apoCIII hyperactivates the CaV-channel through scavenger-receptor class BI (SR-BI)/β1 integrin-dependent co-activation of protein kinase A (PKA) and proto-oncogene tyrosine-protein kinase Src (Src) [84].

The increase in Ca^2+^-channel activity was seen in primary β-cells and β-cell lines, but also in non-β cells, indicating that the observed effects could be of interest not only for β-cells, but as well for cells in other tissues involved in diabetes complications [20,81,85].

In the β-cell line INS-1E, it was demonstrated that elevated levels of apoCIII induced apoptosis by activating the mitogen activated protein kinase (MAPK) p38 and the extracellular signal-regulated kinases 1/2 (ERK_1/2_). If cells were exposed to the L-type Ca^2+^ channel blocker nimodipine, prior to apoCIII, these effects were prevented [86].

Changes in [Ca^2+^]_i_ are playing a major role for the stimulus-secretion coupling leading to secretion of insulin from the β-cells, and the apoCIII-mediated hyperactivation of the voltage-gated Ca^2+^ channels resulted in apoptosis that could be prevented by a Ca^2+^-channel blocker [20,81]. There is a study where they used multiple doses of streptozotocin (STZ) to induce insulin-deficient diabetes in mice and on the fifth and last day of the STZ treatment they started to give verapamil, a Ca^2+^-channel blocker, in the drinking water. The control mice became diabetic, while those given verapamil remained normoglycemic. Immunohistochemistry revealed that in pancreases from the verapamil-treated mice, there were normal insulin containing islets, while in the only STZ-treated mice the islets were destroyed [87]. Although there are no data on apoCIII in this study, it shows the importance of [Ca^2+^]_i_.

Changes in [Ca^2+^]_i_, upon depolarization, were measured in β-cells incubated overnight with sera from children and adults with T1D, first-degree relatives and healthy controls from Finland, Sweden and Miami, FL, USA. Around 30% of the tested sera from T1D patients and first-degree relatives interfered with intracellular Ca^2+^ handling. This effect was not correlated, as mentioned in Section 5, to the presence of autoantibodies, neither to ethnic background, age or gender [80].

### 6.3. Cytokines

When islets from neonatal rats were incubated with the islet cytotoxic cytokines, IL-1β and interferon-γ, to mimic the intraislet inflammatory milieu seen in T1D, the addition of apoCIII to the incubation medium provided protection against apoptosis by degradation of the inhibitor of κΒ (IκΒ) and stimulation of the phosphorylation of survival serine-threonine kinase Akt [67]. Initially, these data seemed contradictory to the data on primary β-cells from adult animals and β-cell lines where apoCIII induced apoptosis. However, the explanation to this discrepancy may be that the levels of apoCIII in neonatal islets are very low and that the addition of the apolipoprotein to the medium with cytokines increased it to levels within the normal range [88]. This is in line with the observations in humans and rodents that not only high, but also too-low, levels of apoCIII are harmful to [89,90].

### 6.4. In Vivo Effects

In the diabetes-prone Biobreeding (DPBB) rat, which develops human-like T1D within a narrow time window of around 60 days [91,92,93], the onset of diabetes was prolonged when apoCIII was lowered during 28 days of the prediabetic phase (from 12 to 40 days of age), when the rats were not insulin deficient and had normal blood glucose levels [93]. These data indicate that other mechanisms mediated by apoCIII can be involved in the development of T1D before the β-cells are destroyed, resulting in a lack of insulin and upregulation of the gene.

The association between T1D and haplotypes within the apoCIII gene has been tested [94]. DNA was collected from 584 T1D patients and 591 control subjects. The samples were genotyped for six single nucleotide polymorphisms in the apoCIII gene (C-641A, C-482T, T-455C, C1100T, C3175G, and T3206G). Two alleles of a haplotype block in the promotor region, containing an insulin response element, were identified to be associated with T1D. The frequency of the A-T-C-C allele was higher, while that of the C-C-T-C allele was reduced, in T1D. Based on these findings a model of the etiology of T1D was proposed by the authors. A haplotype block that includes genetic variants within the regulatory region of the apoCIII promoter results in increased levels of apoCIII and β-cell apoptosis. The progressive reduction of β-cells reduces insulin secretion and further increases apoCIII by the lack of down-regulation of the gene expression by the negative insulin response element. The vicious cycle continues until onset of TID [94].

Many studies have confirmed that elevated apoCIII confer increased risk of macrovascular diseases [61,79,95,96,97,98,99,100,101]. In normolipidemic subjects with T1D, higher circulating levels of apoCIII are associated with changes in subclasses of lipoproteins and an increased risk of CVD [102]. Furthermore, an independent positive association between levels of apoCIII and microvascular complications has been demonstrated in patients with T1D [103].

## 7. Concluding Remarks

T1D, previously called juvenile diabetes, is a disease that can be diagnosed at any age, although the onset is more common at younger ages. It belongs to the autoimmune diseases and it is the vital insulin-secreting pancreatic β-cells that are destroyed. This process of destruction occurs in genetically susceptible individuals during interaction with an immune system that does not distinguish foreign tissue from own tissue, and one or more environmental factors. Although much effort has been made to try to identify underlying mechanisms, we still lack the knowledge of how to prevent or stop ongoing-β-cell destruction.

**Figure 2:**
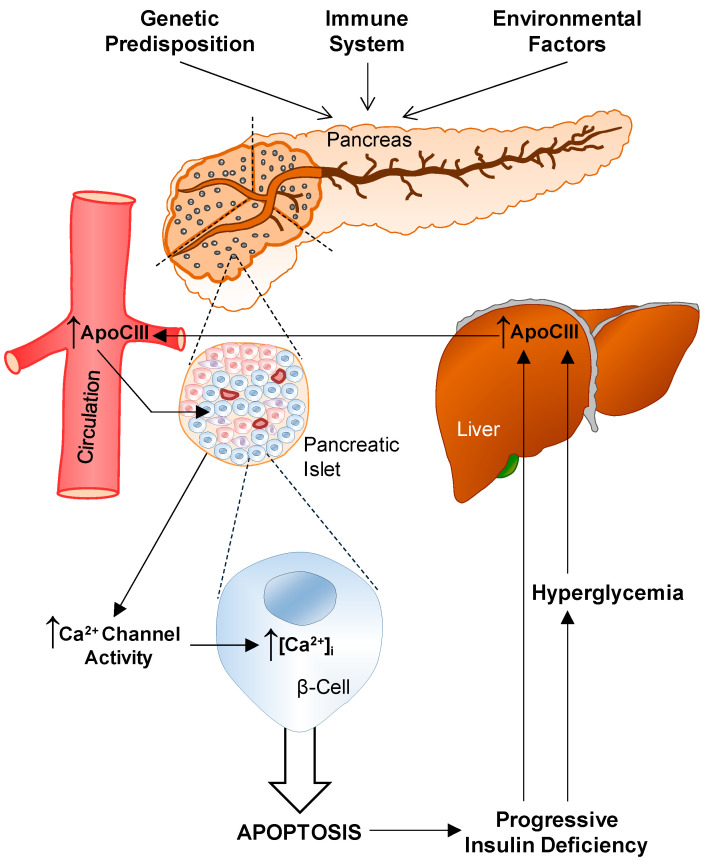
A schematic overview of factors involved in the development of type-1 diabetes (T1D). The background rests on three pillars: genetic predisposition, a dysfunctional immune system and environmental factors. The autoimmune attack on the pancreatic islet β-cells starts. ApoCIII increases the activity of voltage-gated Ca^2+^-channels, thereby increasing [Ca^2+^]_i_ resulting in apoptosis. The progressive decrease in insulin and rise in glucose in the blood diminish the inhibition of the apoCIII gene and the increase in apoCIII hyperactivates Ca^2+^-channels, and a vicious circle is established.

The discovery that healthy β-cells undergo apoptosis if they are exposed to serum from T1D patients and that the responsible serum factor was identified to be apoCIII has created a new and exciting field for investigations (Figure 2).

ApoCIII is an interesting small protein that, for many years, has been known to be a risk factor for CVD [55,56,57] and, in diabetes, most of the complications are related to vascular changes. There are studies on CVD in patients with T1D that show an association with apoCIII [79,102,103]. In the prospective Coronary Artery Calcification Study in Type 1 Diabetes (CACTI), elevated serum apoCIII was observed to be a risk factor for CVD and, although not independent from Tgs, it was a stronger predictor than Tgs [79]. An accumulation of atherogenic lipoproteins in the artery wall, promoted by the increased levels of apoCIII, was suggested as a possible underlying mechanism [79]. When carotid intima-media thickness was measured cross-sectionally and prospectively in subjects with T1D, it pointed to an adverse association to apoCIII [104]. Data from humans reveal that HDL containing apoCIII no longer acts as the “good protective cholesterol”, but instead is related to an increased risk of atherogenesis and diabetes, which further confirms the complexity of this apolipoprotein [105,106].

Several studies have concluded that individuals with mutations in the apoCIII gene, which results in life-long lower levels of the apolipoprotein, are healthier with a favorable pattern of lipoproteins, increased insulin sensitivity, lower incidence of hypertension and they live longer [107,108,109,110]. These data are important since they emphasize that lower than what is considered to be normal levels of apoCIII improve health.

In recent years, the focus regarding apoCIII and its effects has expanded, and this broadened perspective includes T1D. As lowering of apoCIII during a period of the prediabetic phase in the BB rat animal model for T1D delayed the time to onset; it is of interest to investigate whether it is possible, by prolonging the treatment, to prevent the disease. So far, antisense against apoCIII has been used to decrease the lipoprotein, but there are other options such as siRNAs and monoclonal antibodies that can be tested. Furthermore, there is a need to investigate if there is a link to the autoimmune attack against the β-cells and also if viral infections related to T1D involve changes in apoCIII.

The complexity behind the development of T1D is challenging and we need to find the pieces lacking in the jigsaw puzzle to be able to understand the multifaceted pathogenesis of this devastating disease.

## Data Availability

Data sharing not applicable.

## Abbreviations

apoCIII	Apolipoprotein CIII
BB	Biobreeding rat
C/EBPδ/NF-IL6-β	CAAT enhancer-binding protein δ/nuclear factor/IL6- β
CVD	Cardiovascular disease
CaV	Voltage-gated Ca^2+^-channels
ChREBP	Carbohydrate response element–binding protein
DAMPs	Danger-associated molecular patterns
DPBB	Diabetes-prone BB rat
EC	Endothelial cells
ERK_1/2_	Extracellular signal-regulated kinases 1/2
Foxo1	Forkhead box O1
G-banding	Giemsa-banding
HDL	High-density lipoprotein
HNF-4α	Hepatocyte nuclear receptor-4α
HLA	Human leukocyte antigens
ICAM-1	Intracellular adhesion molecule
IL	Interleukin
IDL	Intermediate-density lipoproteins
IRE	Insulin/phorbol ester responsive element
IκΒ	Inhibitor of κΒ
LDL	Low-density lipoprotein
LDLR	LDL receptor
LPL	Lipoprotein lipase
LRP1	LDL-related protein 1
LXR	Liver X receptors
MAPK	Mitogen activated protein kinase
NF-κB	Nuclear factor kappa-B
NLRP3	Nod-like pyrin domain-containing 3
PAMPs	Pathogen-associated molecular patterns
PK	Pyruvate kinase
PKA	Protein kinase A
PKCα	Protein kinase C α
SCIMP	SLP adaptor and CSK-interacting membrane protein
SLE	Systemic lupus erythematosus
SR-BI	Scavenger-receptor class BI
Src	Proto-oncogene tyrosine-protein kinase Src
T1D	Type-1 diabetes
T2D	Type-2 diabetes
TEDDY	The Environmental Determinants of Diabetes in the Young
Tgs	Triglycerides
TNF-α	Tumor necrosis factor α
TRL	Triglyceride-rich lipoprotein
VCAM-1	Vascular cell adhesion molecule-1
VLDL	Very low-density lipoproteins
[Ca^2+^]_i_	Cytoplasmic-free Ca^2+^ concentration

## References

[1] Barrett J.C., Clayton D.G., Concannon P., Akolkar B., Cooper J.D., Erlich H.A., Julier C., Morahan G., Nerup J., Nierras C. (2009). Type 1 Diabetes Genetics Consortium. Genome-wide association study and meta-analysis find that over 40 loci affect risk of type 1 diabetes. Nat. Genet..

[2] Bluestone J.A., Herold K., Eisenbarth G. (2010). Genetics, pathogenesis and clinical interventions in type 1 diabetes. Nature.

[3] Santos D.C., Porto L.C., Oliveira R.V., Secco D., Hanhoerderster L., Pizarro M.H., Barros B.S.V., Mello L.G.N., Muniz L.H., Silva D.A. (2020). HLA class II genotyping of admixed Brazilian patients with type 1 diabetes according to self-reported color/race in a nationwide study. Sci. Rep..

[4] Dayan C.M., Korah M., Tatovic D., Bundy B.N., Herold K.C. (2019). Changing the landscape for type 1 diabetes: The first step to prevention. Lancet.

[5] Steck A.K., Vehik K., Bonifacio E., Lernmark A., Ziegler A.G., Hagopian W.A., She J., Simell O., Akolkar B., Krischer J. (2015). TEDDY Study Group. Predictors of Progression from the Appearance of Islet Autoantibodies to Early Childhood Diabetes: The Environmental Determinants of Diabetes in the Young (TEDDY). Diabetes Care.

[6] Rewers M., Ludvigsson J. (2016). Environmental risk factors for type 1 diabetes. Lancet.

[7] Brown W.V., Levy R.I., Fredrickson D.S. (1969). Studies of the proteins in human plasma very low density lipoproteins. J. Biol. Chem..

[8] Brewer H.B., Shulman R., Herbert P., Ronan R., Wehrly K. (1974). The complete amino acid sequence of alanine apolipoprotein (apoC-3), and apolipoprotein from human plasma very low density lipoproteins. J. Biol. Chem..

[9] Zannis V.I., Cole F.S., Jackson C.L., Kurnit D.M., Karathanasis S.K. (1985). Distribution of apolipoprotein A-I, C-II, C-III, and E mRNA in fetal human tissues. Time-dependent induction of apolipoprotein E mRNA by cultures of human monocyte-macrophages. Biochemistry.

[10] Reue K., Leff T., Breslow J.L. (1988). Human apolipoprotein CIII gene expression is regulated by positive and negative cis-acting elements and tissue-specific protein factors. J. Biol. Chem..

[11] Ogami K., Hadzopoulou-Cladaras M., Cladaras C., Zannis V.I. (1990). Promoter elements and factors required for hepatic and intestinal transcription of the human ApoCIII gene. J. Biol. Chem..

[12] West G., Rodia C., Li D., Johnson Z., Dong H., Kohan A.B. (2017). Key differences between apoC-III regulation and expression in intestine and liver. Biochem. Biophys. Res. Commun..

[13] Vaith P., Assmann G., Uhlenbruck G. (1978). Characterization of the oligosaccharide side chain of apolipoprotein C-III from human plasma very low density lipoproteins. Biochim. Biophys. Acta.

[14] Ito Y., Breslow J.L., Chait B.T. (1989). Apolipoprotein C-III0 lacks carbohydrate residues: Use of mass spectrometry to study apolipoprotein structure. J. Lipid. Res..

[15] Kashyap M.L., Srivastava L.S., Hynd B.A., Gartside P.S., Perisutti G. (1981). Quantitation of human apolipoprotein C-III and its subspecie by radioimmunoassay and analytical isoelectric focusing: Abnormal plasma triglyceride-rich lipoprotein apolipoprotein C-III subspecie concentrations in hypertriglyceridemia. J. Lipid. Res..

[16] Roghani A., Zannis V.I. (1988). Mutagenesis of the glycosylation site of human ApoCIII. O-linked glycosylation is not required for ApoCIII secretion and lipid binding. J. Biol. Chem..

[17] Mauger J.F., Couture P., Bergeron N., Lamarche B. (2006). Apolipoprotein C-III isoforms: Kinetics and relative implication in lipid metabolism. J. Lipid. Res..

[18] Kegulian N.C., Ramms B., Horton S., Trenchevska O., Nedelkov D., Graham M.J., Lee R.G., Esko J.D., Yassine H.N., Gordts P.L.S.M. (2019). ApoC-III Glycoforms Are Differentially Cleared by Hepatic TRL (Triglyceride-Rich Lipoprotein) Receptors. Arterioscler. Thromb. Vasc. Biol..

[19] Olivieri O., Chiariello C., Martinelli N., Castagna A., Speziali G., Girelli D., Pizzolo F., Bassi A., Cecconi D., Robotti E. (2018). Sialylated isoforms of apolipoprotein C-III and plasma lipids in subjects with coronary artery disease. Clin. Chem. Lab. Med..

[20] Juntti-Berggren L., Refai E., Appelskog I., Andersson M., Imreh G., Dekki N., Uhles S., Yu L., Griffiths W.J., Zaitsev S. (2004). Apolipoprotein CIII promotes Ca^2+^-dependent-cell death in type 1 diabetes. Proc. Natl. Acad. Sci. USA.

[21] Alaupovic P. (1996). Significance of apolipoproteins for structure, function, and classification of plasma lipoproteins. Methods Enzymol..

[22] Khoo C., Campos H., Judge H., Sacks F.M. (1999). Effects of estrogenic oral contraceptives on the lipoprotein B particle system defined by apolipoproteins E and C-III content. J. Lipid. Res..

[23] Jong M.C., Hofker M.H., Havekes L.M. (1999). Role of ApoCs in lipoprotein metabolism: Functional differences between ApoC1, ApoC2, and ApoC3. Arterioscler. Thromb. Vasc. Biol..

[24] Campos H., Perlov D., Khoo C., Sacks F.M. (2001). Distinct patterns of lipoproteins with apoB defined by presence of apoE or apoC-III in hypercholesterolemia and hypertriglyceridemia. J. Lipid. Res..

[25] Taskinen M.R., Borén J. (2016). Why Is Apolipoprotein CIII Emerging as a Novel Therapeutic Target to Reduce the Burden of Cardiovascular Disease?. Curr. Atheroscler. Rep..

[26] Zvintzou E., Lhomme M., Chasapi S., Filou S., Theodoropoulos V., Xapapadaki E., Kontush A., Spyroulias G., Tellis C.C., Tselepis A.D. (2017). Pleiotropic effects of apolipoprotein C3 on HDL functionality and adipose tissue metabolic activity. J. Lipid. Res..

[27] Ginsberg H.N., Le N.A., Goldberg I.J., Gibson J.C., Rubinstein A., Wang-Iverson P., Norum R., Brown W.V. (1986). Apolipoprotein B metabolism in subjects with deficiency of apolipoproteins CIII and AI. Evidence that apolipoprotein CIII inhibits catabolism of triglyceride-rich lipoproteins by lipoprotein lipase in vivo. J. Clin. Investig..

[28] Ebara T., Ramakrishnan R., Steiner G., Shachter N.S. (1997). Chylomicronemia due to apolipoprotein CIII overexpression in apolipoprotein E-null mice. Apolipoprotein CIII-induced hypertriglyceridemia is not mediated by effects on apolipoprotein E. J. Clin. Investig..

[29] Lambert D.A., Smith L.C., Pownall H., Sparrow J.T., Nicolas J.P., Gotto A.M. (2000). Hydrolysis of phospholipids by purified milk lipoprotein lipase. Effect of apoprotein CII, CIII, A and E, and synthetic fragments. Clin. Chim. Acta.

[30] Larsson M., Vorrsjö E., Talmud P., Lookene A., Olivecrona G. (2013). Apolipoproteins C-I and C-III inhibit lipoprotein lipase activity by displacement of the enzyme from lipid droplets. J. Biol. Chem..

[31] Sacks F.M. (2015). The crucial roles of apolipoproteins E and C-III in apoB lipoprotein metabolism in normolipidemia and hypertriglyceridemia. Curr. Opin. Lipidol..

[32] Gordts P.L., Nock R., Son N.H., Ramms B., Lew I., Gonzales J.C., Thacker B.E., Basu D., Lee R.G., Mullick A.E. (2016). ApoC-III inhibits clearance of triglyceride-rich lipoproteins through LDL family receptors. J. Clin. Investig..

[33] Karathanasis S.K., McPherson J., Zannis V.I., Breslow J.L. (1983). Linkage of human apolipoproteins A-I and C-III genes. Nature.

[34] Bruns G.A., Karathanasis S.K., Breslow J.L. (1984). Human apolipoprotein A-I--C-III gene complex is located on chromosome 11. Arteriosclerosis.

[35] Karathanasis S.K. (1985). Apolipoprotein multigene family: Tandem organization of human apolipoprotein AI, CIII, and AIV genes. Proc. Natl. Acad. Sci. USA.

[36] Kan H.Y., Georgopoulos S., Zannis V. (2000). A hormone response element in the human apolipoprotein CIII (ApoCIII) enhancer is essential for intestinal expression of the ApoA-I and ApoCIII genes and contributes to the hepatic expression of the two linked genes in transgenic mice. J. Biol. Chem..

[37] Guardiola M., Oliva I., Guillaumet A., Martín-Trujillo Á., Rosales R., Vallvé J.C., Sabench F., Del Castillo D., Zaina S., Monk D. (2014). Tissue-specific DNA methylation profiles regulate liver-specific expression of the APOA1/C3/A4/A5 cluster and can be manipulated with demethylating agents on intestinal cells. Atherosclerosis.

[38] Altomonte J., Cong L., Harbaran S., Richter A., Xu J., Meseck M., Dong H.H. (2004). Foxo1 mediates insulin action on apoC-III and triglyceride metabolism. J. Clin. Investig..

[39] Caron S., Verrijken A., Mertens I., Samanez C.H., Mautino G., Haas J.T., Duran-Sandoval D., Prawitt J., Francque S., Vallez E. (2011). Transcriptional activation of apolipoprotein CIII expression by glucose may contribute to diabetic dyslipidemia. Arterioscler. Thromb. Vasc. Biol..

[40] Lacorte J.M., Beigneux A., Parant M., Chambaz J. (1997). Repression of apoC-III gene expression by TNFalpha involves C/EBPdelta/NF-IL6beta via an IL-1 independent pathway. FEBS Lett..

[41] Lacorte J.M., Ktistaki E., Beigneux A., Zannis V.I., Chambaz J., Talianidis I. (1997). Activation of CAAT enhancer-binding protein delta (C/EBPdelta) by interleukin-1 negatively influences apolipoprotein C-III expression. J. Biol. Chem..

[42] Chen M., Breslow J.L., Li W., Leff T. (1994). Transcriptional regulation of the apoC-III gene by insulin in diabetic mice: Correlation with changes in plasma triglyceride levels. J. Lipid. Res..

[43] Li W.W., Dammerman M.M., Smith J.D., Metzger S., Breslow J.L., Leff T. (1995). Common genetic variation in the promoter of the human apo CIII gene abolishes regulation by insulin and may contribute to hypertriglyceridemia. J. Clin. Investig..

[44] Borén J., Packard C.J., Taskinen M.R. (2020). The Roles of ApoC-III on the Metabolism of Triglyceride-Rich Lipoproteins in Humans. Front. Endocrinol..

[45] Nakae J., Kitamura T., Silver D.L., Accili D. (2001). The forkhead transcription factor Foxo1 (Fkhr) confers insulin sensitivity onto glucose-6-phosphatase expression. J. Clin. Investig..

[46] Nakae J., Biggs W.H., Kitamura T., Cavenee W.K., Wright C.V., Arden K.C., Accili D. (2002). Regulation of insulin action and pancreatic beta-cell function by mutated alleles of the gene encoding forkhead transcription factor Foxo1. Nat. Genet..

[47] Shih H.M., Liu Z., Towle H.C. (1995). Two CACGTG motifs with proper spacing dictate the carbohydrate regulation of hepatic gene transcription. J. Biol. Chem..

[48] Yamashita H., Takenoshita M., Sakurai M., Bruick R.K., Henzel W.J., Shillinglaw W., Arnot D., Uyeda K. (2001). A glucose-responsive transcription factor that regulates carbohydrate metabolism in the liver. Proc. Natl. Acad. Sci. USA.

[49] Iizuka K., Bruick R.K., Liang G., Horton J.D., Uyeda K. (2004). Deficiency of carbohydrate response element-binding protein (ChREBP) reduces lipogenesis as well as glycolysis. Proc. Natl. Acad. Sci. USA.

[50] Towle H.C. (2005). Glucose as a regulator of eukaryotic gene transcription. Trends Endocrinol. Metab..

[51] Adamson A.W., Suchankova G., Rufo C., Nakamura M.T., Teran-Garcia M., Clarke S.D., Gettys T.W. (2006). Hepatocyte nuclear factor-4alpha contributes to carbohydrate-induced transcriptional activation of hepatic fatty acid synthase. Biochem. J..

[52] Cha J.Y., Repa J.J. (2007). The liver X receptor (LXR) and hepatic lipogenesis. The carbohydrate-response element-binding protein is a target gene of LXR. J. Biol. Chem..

[53] Kardassis D., Tzameli I., Hadzopoulou-Cladaras M., Talianidis I., Zannis V. (1997). Distal apolipoprotein C-III regulatory elements F to J act as a general modular enhancer for proximal promoters that contain hormone response elements. Synergism between hepatic nuclear factor-4 molecules bound to the proximal promoter and distal enhancer sites. Arterioscler. Thromb. Vasc. Biol..

[54] Gruber P.J., Torres-Rosado A., Wolak M.L., Leff T. (1994). Apo CIII gene transcription is regulated by a cytokine inducible NF-kappa B element. Nucleic. Acids. Res..

[55] Kawakami A., Aikawa M., Alcaide P., Luscinskas F.W., Libby P., Sacks F.M. (2006). Apolipoprotein CIII induces expression of vascular cell adhesion molecule-1 in vascular endothelial cells and increases adhesion of monocytic cells. Circulation.

[56] Kawakami A., Aikawa M., Libby P., Alcaide P., Luscinskas F.W., Sacks F.M. (2006). Apolipoprotein CIII in apolipoprotein B lipoproteins enhances the adhesion of human monocytic cells to endothelial cells. Circulation.

[57] Lee S.J., Campos H., Moye L.A., Sacks F.M. (2003). LDL containing apolipoprotein CIII is an independent risk factor for coronary events in diabetic patients. Arterioscler. Thromb. Vasc. Biol..

[58] Katzmann J.L., Werner C.M., Stojakovic T., März W., Scharnagl H., Laufs U. (2020). Apolipoprotein CIII predicts cardiovascular events in patients with coronary artery disease: A prospective observational study. Lipids Health Dis..

[59] Rocha N.A., East C., Zhang J., McCullough P.A. (2017). ApoCIII as a Cardiovascular Risk Factor and Modulation by the Novel Lipid-Lowering Agent Volanesorsen. Curr. Atheroscler. Rep..

[60] Kawakami A., Aikawa M., Nitta N., Yoshida M., Libby P., Sacks F.M. (2007). Apolipoprotein CIII-induced THP-1 cell adhesion to endothelial cells involves pertussis toxin-sensitive G protein- and protein kinase C alpha-mediated nuclear factor-kappaB activation. Arterioscler. Thromb. Vasc. Biol..

[61] Hiukka A., Ståhlman M., Pettersson C., Levin M., Adiels M., Teneberg S., Leinonen E.S., Hultén L.M., Wiklund O., Oresic M. (2009). ApoCIII-enriched LDL in type 2 diabetes displays altered lipid composition, increased susceptibility for sphingomyelinase, and increased binding to biglycan. Diabetes.

[62] Guo H., Callaway J.B., Ting J.P. (2015). Inflammasomes: Mechanism of action, role in disease, and therapeutics. Nat. Med..

[63] Zewinger S., Reiser J., Jankowski V., Alansary D., Hahm E., Triem S., Klug M., Schunk S.J., Schmit D., Kramann R. (2020). Apolipoprotein C3 induces inflammation and organ damage by alternative inflammasome activation. Nat. Immunol..

[64] Paschou S.A., Papadopoulou-Marketou N., Chrousos G.P., Kanaka-Gantenbein C. (2018). On type 1 diabetes mellitus pathogenesis. Endocr. Connect..

[65] Morgan P.E., Sturgess A.D., Hennessy A., Davies M.J. (2007). Serum protein oxidation and apolipoprotein CIII levels in people with systemic lupus erythematosus with and without nephritis. Free Radic. Res..

[66] Araújo D.M., Rodrigues C.E.M., Gonçalves N.G.G., Rabelo-Júnior C.N., Lobo M.D.P., Moreira R.A., Monteiro-Moreira A.C.O. (2020). Proteins Involved in the Induction of Procoagulant Activity and Autoimmune Response in Patients with Primary Antiphospholipid Syndrome. Clin. Appl. Thromb. Hemost..

[67] Størling J., Juntti-Berggren L., Olivecrona G., Prause M.C., Berggren P.O., Mandrup-Poulsen T. (2011). Apolipoprotein CIII reduces proinflammatory cytokine-induced apoptosis in rat pancreatic islets via the Akt prosurvival pathway. Endocrinology.

[68] Frostegård J. (2005). SLE, atherosclerosis and cardiovascular disease. J. Intern. Med..

[69] Esdaile J.M., Abrahamowicz M., Grodzicky T., Li Y., Panaritis C., du Berger R., Côte R., Grover S.A., Fortin P.R., Clarke A.E. (2001). Traditional Framingham risk factors fail to fully account for accelerated atherosclerosis in systemic lupus erythematosus. Arthritis Rheum..

[70] Briones E.R., Mao S.J., Palumbo P.J., O’Fallon W.M., Chenoweth W., Kottke B.A. (1984). Analysis of plasma lipids and apolipoproteins in insulin-dependent and noninsulin-dependent diabetics. Metabolism.

[71] Joven J., Vilella E., Costa B., Turner P.R., Richart C., Masana L. (1989). Concentrations of lipids and apolipoproteins in patients with clinically well-controlled insulin-dependent and non-insulin-dependent diabetes. Clin. Chem..

[72] Stewart M.W., Laker M.F., Alberti K.G. (1994). The contribution of lipids to coronary heart disease in diabetes mellitus. J. Intern. Med. Suppl..

[73] Bren N.D., Rastogi A., Kottke B.A. (1993). Quantification of human plasma apolipoproteins C-I, C-II, and C-III by radioimmunoassays. Mayo Clin. Proc..

[74] Nestel P.J., Fidge N.H. (1982). Apoprotein C metabolism in man. Adv. Lipid Res..

[75] Blackett P., Sarale D.C., Fesmire J., Harmon J., Weech P., Alaupovic P. (1988). Plasma apolipoprotein C-III levels in children with type I diabetes. South Med. J..

[76] Al Muhtaseb N., al Yousuf A., Bajaj J.S. (1992). Apolipoprotein A-I, A-II, B, C-II, and C-III in children with insulin-dependent diabetes mellitus. Pediatrics.

[77] Manzato E., Zambon A., Lapolla A., Zambon S., Braghetto L., Crepaldi G., Fedele D. (1993). Lipoprotein abnormalities in well-treated type II diabetic patients. Diabetes Care.

[78] Reverter J.L., Sentí M., Rubiés-Prat J., Lucas A., Salinas I., Pizarro E., Pedro-Botet J., Sanmartí A. (1993). Lipoprotein composition in the insulin-deficient non-acidotic phase of type I diabetic patients and early evolution after the start of insulin therapy. Clin. Chim. Acta.

[79] Kanter J.E., Shao B., Kramer F., Barnhart S., Shimizu-Albergine M., Vaisar T., Graham M.J., Crooke R.M., Manuel C.R., Haeusler R.A. (2019). Increased apolipoprotein C3 drives cardiovascular risk in type 1 diabetes. J. Clin. Investig..

[80] Dekki N., Nilsson R., Norgren S., Rössner S.M., Appelskog I., Marcus C., Simell O., Pugliese A., Alejandro R., Ricordi C. (2007). Type 1 diabetic serum interferes with pancreatic beta-cell Ca^2+^-handling. Biosci. Rep..

[81] Juntti-Berggren L., Larsson O., Rorsman P., Ammälä C., Bokvist K., Wåhlander K., Nicotera P., Dypbukt J., Orrenius S., Hallberg A. (1993). Increased activity of L-type Ca2+ channels exposed to serum from patients with type I diabetes. Science.

[82] Yang S.N., Shi Y., Yang G., Li Y., Yu J., Berggren P.-O. (2014). Ionic mechanisms in pancreatic β cell signaling. Cell Mol. Life Sci..

[83] Yang G., Shi Y., Yu J., Li Y., Yu L., Welling A., Hofmann F., Striessnig J., Juntti-Berggren L., Berggren P.O. (2015). CaV1.2 and CaV1.3 channel hyperactivation in mouse islet β cells exposed to type 1 diabetic serum. Cell Mol. Life Sci..

[84] Shi Y., Yang G., Yu J., Yu L., Westenbroek R., Catterall W.A., Juntti-Berggren L., Berggren P.O., Yang S.N. (2014). Apolipoprotein CIII hyperactivates β cell CaV1 channels through SR-BI/β1 integrin-dependent coactivation of PKA and Src. Cell Mol. Life Sci..

[85] Refai E., Dekki N., Yang S.N., Imreh G., Cabrera O., Yu L., Yang G., Norgren S., Rössner S.M., Inverardi L. (2005). Transthyretin constitutes a functional component in pancreatic β-cell stimulus-secretion coupling. Proc. Natl. Acad. Sci. USA.

[86] Sol E.M., Sundsten T., Bergsten P. (2009). Role of MAPK in apolipoprotein CIII-induced apoptosis in INS-1E cells. Lipids Health Dis..

[87] Xu G., Chen J., Jing G., Shalev A. (2012). Preventing β-cell loss and diabetes with calcium channel blockers. Diabetes.

[88] Åvall K., Berggren P.O., Juntti-Berggren L. (2018). The yin and yang of apolipoprotein CIII. Diabetes Metab..

[89] Norum R.A., Lakier J.B., Goldstein S., Angel A., Goldberg R.B., Block W.D., Noffze D.K., Dolphin P.J., Edelglass J., Bogorad D.D. (1982). Familial deficiency of apolipoproteins A-I and C-III and precocious coronary-artery disease. N. Engl. J. Med..

[90] Duivenvoorden I., Teusink B., Rensen P.C., Romijn J.A., Havekes L.M., Voshol P.J. (2005). Apolipoprotein C3 deficiency results in diet-induced obesity and aggravated insulin resistance in mice. Diabetes.

[91] Nakhooda A.F., Like A.A., Chappel C.I., Murray F.T., Marliss E.B. (1977). The spontaneously diabetic Wistar rat. Metabolic and morphologic studies. Diabetes.

[92] Nakhooda A.F., Like A.A., Chappel C.I., Wei C.N., Marliss E.B. (1978). The spontaneously diabetic Wistar rat (the "BB" rat). Studies prior to and during development of the overt syndrome. Diabetologia.

[93] Holmberg R., Refai E., Höög A., Crooke R.M., Graham M., Olivecrona G., Berggren P.O., Juntti-Berggren L. (2011). Lowering apolipoprotein CIII delays onset of type 1 diabetes. Proc. Natl. Acad. Sci. USA.

[94] Hokanson J.E., Kinney G.L., Cheng S., Erlich H.A., Kretowski A., Rewers M. (2006). Susceptibility to type 1 diabetes is associated with ApoCIII gene haplotypes. Diabetes.

[95] Blankenhorn D.H., Alaupovic P., Wickham E., Chin H.P., Azen S.P. (1990). Prediction of angiographic change in native human coronary arteries and aortocoronary bypass grafts. Lipid and nonlipid factors. Circulation.

[96] Gervaise N., Garrigue M.A., Lasfargues G., Lecomte P. (2000). Triglycerides, apo C3 and Lp B:C3 and cardiovascular risk in type II diabetes. Diabetologia.

[97] Hodis H.N., Mack W.J., Azen S.P., Alaupovic P., Pogoda J.M., LaBree L., Hemphill L.C., Kramsch D.M., Blankenhorn D.H. (1994). Triglyceride- and cholesterol-rich lipoproteins have a differential effect on mild/moderate and severe lesion progression as assessed by quantitative coronary angiography in a controlled trial of lovastatin. Circulation.

[98] Koren E., Corder C., Mueller G., Centurion H., Hallum G., Fesmire J., McConathy W.D., Alaupovic P. (1996). Triglyceride enriched lipoprotein particles correlate with the severity of coronary artery disease. Atherosclerosis.

[99] Krauss R.M., Kesäniemi Y.A. (1994). Cardiovascular disease and hyperlipidaemia. Curr. Opin. Lipidol..

[100] Luc G., Fievet C., Arveiler D., Evans A.E., Bard J.M., Cambien F., Fruchart J.C., Ducimetiere P. (1996). Apolipoproteins C-III and E in apoB- and non-apoB-containing lipoproteins in two populations at contrasting risk for myocardial infarction: The ECTIM study. Etude Cas Témoins sur ’Infarctus du Myocarde. J. Lipid. Res..

[101] Sacks F.M., Alaupovic P., Moye L.A., Cole T.G., Sussex B., Stampfer M.J., Pfeffer M.A., Braunwald E. (2000). VLDL, apolipoproteins B, CIII, and E, and risk of recurrent coronary events in the Cholesterol and Recurrent Events (CARE) trial. Circulation.

[102] Klein R.L., McHenry M.B., Lok K.H., Hunter S.J., Le N.A., Jenkins A.J., Zheng D., Semler A.J., Brown W.V., DCCT/EDIC Research Group (2004). Apolipoprotein C-III protein concentrations and gene polymorphisms in type 1 diabetes: Associations with lipoprotein subclasses. Metabolism.

[103] Klein R.L., McHenry M.B., Lok K.H., Hunter S.J., Le N.A., Jenkins A.J., Zheng D., Semler A., Page G., Brown W.V. (2005). DCCT/EDIC Research Group. Apolipoprotein C-III protein concentrations and gene polymorphisms in Type 1 diabetes: Associations with microvascular disease complications in the DCCT/EDIC cohort. J. Diabetes Complicat..

[104] Basu A., Jenkins A.J., Stoner J.A., Zhang Y., Klein R.L., Lopes-Virella M.F., Garvey W.T., Schade D.S., Wood J., Alaupovic P. (2018). Diabetes Control and Complications Trial/Epidemiology of Diabetes Interventions and Complications Research Group. Apolipoprotein-defined lipoprotein subclasses, serum apolipoproteins, and carotid intima-media thickness in T1D. J. Lipid Res..

[105] Aroner S.A., Koch M., Mukamal K.J., Furtado J.D., Stein J.H., Tattersall M.C., McClelland R.L., Jensen M.K. (2018). High-Density Lipoprotein Subspecies Defined by Apolipoprotein C-III and Subclinical Atherosclerosis Measures: MESA (The Multi-Ethnic Study of Atherosclerosis). J. Am. Heart Assoc..

[106] Aroner S.A., Furtado J.D., Sacks F.M., Tsai M.Y., Mukamal K.J., McClelland R.L., Jensen M.K. (2019). Apolipoprotein C-III and its defined lipoprotein subspecies in relation to incident diabetes: The Multi-Ethnic Study of Atherosclerosis. Diabetologia.

[107] Atzmon G., Rincon M., Schechter C.B., Shuldiner A.R., Lipton R.B., Bergman A., Barzilai N. (2006). Lipoprotein genotype and conserved pathway for exceptional longevity in humans. PLoS Biol..

[108] Pollin T.I., Damcott C.M., Shen H., Ott S.H., Shelton J., Horenstein R.B., Post W., McLenithan J.C., Bielak L.F., Peyser P.A. (2008). A null mutation in human APOC3 confers a favorable plasma lipid profile and apparent cardioprotection. Science.

[109] Crosby J., Peloso G.M., Auer P.L., Crosslin D.R., Stitziel N.O., Lange L.A., Lu Y., Tang Z.Z., Zhang H., TG and HDL Working Group of the Exome Sequencing Project, National Heart, Lung, and Blood Institute (2014). Loss-of-function mutations in APOC3, triglycerides, and coronary disease. N. Engl. J. Med..

[110] Jørgensen A.B., Frikke-Schmidt R., Nordestgaard B.G., Tybjærg-Hansen A. (2014). Loss-of-function mutations in APOC3 and risk of ischemic vascular disease. N. Engl. J. Med..

